# Transportin 1 is a major nuclear import receptor of the nitric oxide synthase interacting protein

**DOI:** 10.1016/j.jbc.2023.102932

**Published:** 2023-01-20

**Authors:** Marius Pörschke, Inés Rodríguez-González, Iwan Parfentev, Henning Urlaub, Ralph H. Kehlenbach

**Affiliations:** 1 Department of Molecular Biology, Faculty of Medicine, GZMB, Georg-August-University Göttingen, Göttingen, Germany; 2 Bioanalytical Mass Spectrometry Group, Max Planck Institute for Multidisciplinary Sciences, Göttingen, Germany; 3 Bioanalytics Group, Institute of Clinical Chemistry, University Medical Center Göttingen, Göttingen, Germany

**Keywords:** transportin 1, TNPO1, importin α/β, NOSIP, eNOS, nuclear transport, BSA, bovine serum albumin, cNLS, classic NLS, DMEM, Dulbecco’s modified Eagle’s medium, eNOS, endothelial nitric oxide synthase, FL, full length, GR, glucocorticoid receptor, GST, glutathione-S-transferase, Imp, importin, NLS, nuclear localization signal, NO, nitric oxide, NOSIP, nitric oxide synthase interacting protein, NTR, nuclear transport receptor, RT, room temperature, TNPO1, transportin 1, TPB, transport buffer

## Abstract

The nitric oxide synthase interacting protein (NOSIP), an E3-ubiquitin ligase, is involved in various processes like neuronal development, craniofacial development, granulopoiesis, mitogenic signaling, apoptosis, and cell proliferation. The best-characterized function of NOSIP is the regulation of endothelial nitric oxide synthase activity by translocating the membrane-bound enzyme to the cytoskeleton, specifically in the G2 phase of the cell cycle. For this, NOSIP itself has to be translocated from its prominent localization, the nucleus, to the cytoplasm. Nuclear import of NOSIP was suggested to be mediated by the canonical transport receptors importin α/β. Recently, we found NOSIP in a proteomic screen as a potential importin 13 cargo. Here, we describe the nuclear shuttling characteristics of NOSIP in living cells and *in vitro* and show that it does not interact directly with importin α. Instead, it formed stable complexes with several importins (−β, −7, −β/7, −13, and transportin 1) and was also imported into the nucleus in digitonin-permeabilized cells by these factors. In living HeLa cells, transportin 1 seems to be the major nuclear import receptor for NOSIP. A detailed analysis of the NOSIP-transportin 1 interaction revealed a high affinity and an unusual binding mode, involving the N-terminal half of transportin 1. In contrast to nuclear import, nuclear export of NOSIP seems to occur mostly by passive diffusion. Thus, our results uncover additional layers in the larger process of endothelial nitric oxide synthase regulation.

Nitric oxide (NO) signaling is key to the regulation of many biological processes, including vascular homeostasis, antimicrobial defense, inflammation, wound healing, apoptosis, proliferation, angiogenesis, and neuronal plasticity (1, 2). Endothelial nitric oxide synthase (eNOS) is one of three members of a family of enzymes that uses L-arginine for NO synthesis (3). eNOS is present in endothelial cells, platelets, and cardiac myocytes (3). Its activity is regulated by the intracellular Ca^2+^ concentration. At high Ca^2+^ concentrations, calmodulin binds to eNOS and enhances its activity (for review see (4)). When Ca^2+^ concentrations are low, calmodulin dissociates from eNOS, which then binds to caveolin-1, an inhibitor of eNOS (4). In addition, these interactions can be fine-tuned by several effector proteins like Hsp90, porin, and G protein–coupled receptors (4).

In addition to the regulation of eNOS activity by Ca^2+^, the intracellular localization of the enzyme affects its activity. It has been reported that trafficking of eNOS, which is usually located at the plasma membrane, toward the cytoskeleton or intracellular organelles leads to altered activity. The translocation of eNOS is facilitated by interacting proteins like NOSTRIN (eNOS traffic inducer (5)) or NOSIP (nitric oxide synthase interacting protein). The 34-kDa protein NOSIP was first identified in a yeast two-hybrid screen as an interaction partner of eNOS (6) and was later shown to also bind to a neuronal form of the enzyme (nNOS) (7). NOSIP has been associated with several diseases, like osteoporosis and arteriosclerosis (8), Hirschsprung disease (9) and psychological developmental disorders (10). On a molecular level, NOSIP has been suggested to function as an E3-ubiquitin ligase and to mediate the ubiquitination of the erythropoietin receptor (EpoR) (11) and the protein phosphatase 2A catalytic subunit (PP2Ac) (12). Thereby, NOSIP may play a role in mitogenic signaling *via* the erythropoietin receptor and in proper brain and craniofacial development by the regulation of PP2A activity. Upon binding of NOSIP, eNOS translocates from the plasma membrane to the cytoskeleton, resulting in an indirect downregulation of its enzymatic activity (6, 7, 13, 14).

In many cells and tissues, NOSIP is predominantly nuclear, but the protein has also been detected in the cytoplasm (7, 14, 15). Clearly, its subcellular localization is subject to regulation, as NOSIP shifts from the nucleus to the cytoplasm in the G2 phase of the cell cycle (14). Accordingly, a concomitant downregulation of eNOS activity has been suggested.

NOSIP has previously been shown to contain a nonconventional bipartite nuclear localization signal (NLS; Fig. 1*A*), and the canonical transport receptor importin α was suggested as a mediator of nuclear import (14). Typically, importin α functions as an adapter protein that interacts with proteins containing a classic NLS and also binds importin β, a *bona fide* import receptor. Importin β is a member of a large family of transport receptors that also includes transportin 1 (TNPO1) (16), another prominent import receptor, and CRM1, the major nuclear export receptor (17). All importins and exportins of the importin β superfamily (collectively also known as karyopherins) interact with the GTP-bound form of Ran, a major regulator of nucleocytoplasmic transport (for review see (16)). In nuclear import, binding of RanGTP to an importin leads to the dissociation of the transport complex in the nucleus. In nuclear export, by contrast, RanGTP is required for the formation of a trimeric export complex in the nucleus. Another common feature of importins and exportins is their ability to interact with nucleoporins, components of the nuclear pore complex, thus allowing translocation of transport complexes across the nuclear envelope. Recently, we identified NOSIP as a specific binding partner of the transport receptor importin 13, another member of the importin β superfamily (18). Curiously, importin 13 can function in both nuclear import and nuclear export (19). We therefore decided to investigate the nucleocytoplasmic shuttling of NOSIP in more detail. Indeed, several transport receptors interact specifically with NOSIP in a RanGTP-dependent manner. In HeLa cells and in *in vitro* nuclear import assays, we identified TNPO1 as a major import receptor for NOSIP and showed that the two proteins interact in an unconventional manner. Other importins, including importin β and importin 13, can promote nuclear import of NOSIP as well, whereas importin α may only play a minor role. Nuclear export of NOSIP, on the other hand, seems to occur only by passive diffusion, *i.e.*, independent of transport receptors.

**Figure 1:**
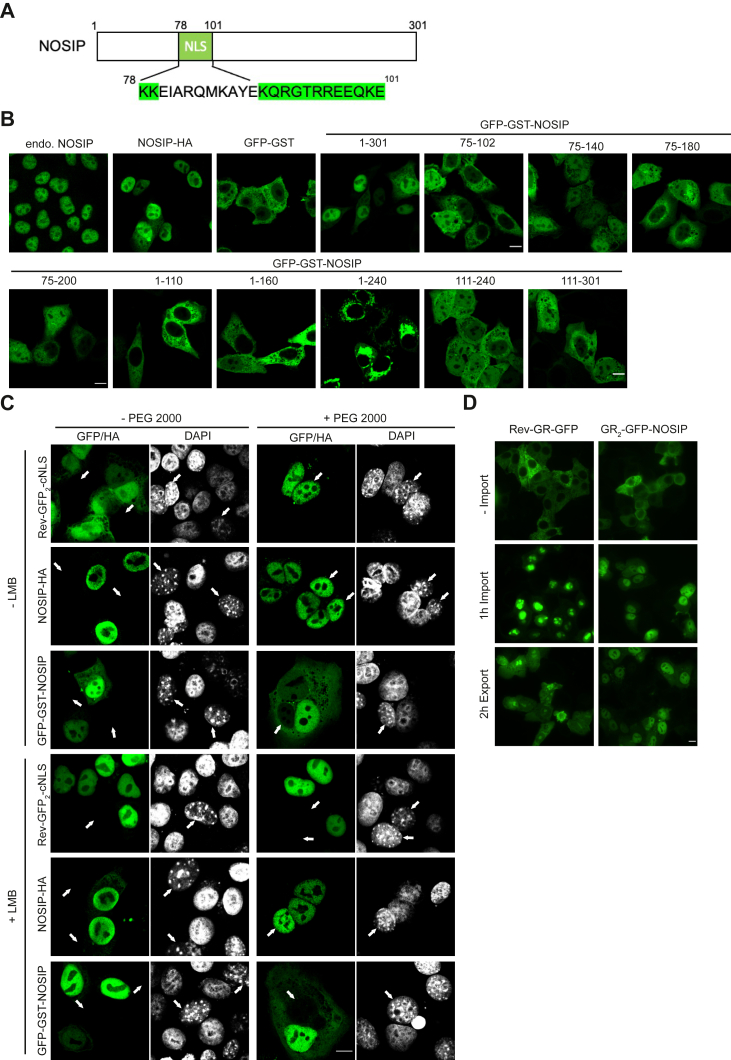
**Active nuclear import and passive export of NOSIP.***A*, schematic representation of NOSIP with its nuclear localization signal (NLS, *green*) as previously suggested (14). *B*, HeLa cells were transfected with constructs coding for HA-or GFP-GST-tagged versions of NOSIP as indicated, and NOSIP was visualized *via* the GFP-tag or by indirect immunofluorescence using antibodies against the HA-tag. Endogenous (endo.) NOSIP was detected using specific antibodies. Cells were analyzed by confocal microscopy. *C*, HeLa cells were transfected with plasmids coding for Rev^47–116^-GFP_2_-cNLS, NOSIP-HA, or GFP-GST-NOSIP as indicated and cocultured with NIH3T3 cells. Three hours prior and after a short treatment with (+PEG2000) or without (−PEG200) polyethylene glycol (PEG), cells were incubated with (+LMB) or without (−LMB) 10 nM LMB and analyzed by confocal microscopy. NIH3T3 nuclei can be differentiated by the DAPI stain (*gray*) from HeLa nuclei and are marked with *arrows*. *D*, HeLa cells were transfected with plasmids coding for GR_2_-GFP-NOSIP or Rev-GR-GFP. Nuclear import was induced by the addition of 5 μM dexamethasone for 1 h (1 h import). For the analysis of nuclear export, cells were then incubated in fresh medium lacking dexamethasone for 2 h (2 h export). Cells were analyzed by fluorescence microscopy. *B–D*, the scale bars represent 10 μm.

## Results

### Active nuclear import of NOSIP

Endogenous NOSIP has previously been shown to localize primarily to the nucleus of cultured cells (14). Likewise, GFP-fusions of NOSIP accumulated in the nucleus, suggesting active nuclear import. In these studies, the classic nuclear import receptor importin α, which functions in concert with importin β, was reported to serve as an import factor for NOSIP. The authors also noted, however, that the identified NLS (amino acids 78–101; Fig. 1*A*) does not match the canonical mono- or bipartite NLS as it can be found in many nuclear proteins. We therefore decided to analyze the nucleocytoplasmic shuttling of NOSIP and its potential nuclear transport receptors in more detail. As described before (14), endogenous NOSIP as well as exogenous, HA-tagged NOSIP was detected in nuclei of HeLa cells (Fig. 1*A*). Being a small protein of 34 kDa, NOSIP (with or without an HA-tag) could passively diffuse through the nuclear pore complex and then be retained in the nucleus upon interaction with a binding partner. We therefore fused the coding sequence of NOSIP to that of a large reporter protein, GFP–glutathione-*S*-transferase (GST), which on its own does not enter the nucleus efficiently (20). The resulting protein, GFP-GST-NOSIP, with a calculated molecular mass of 87 kDa that is above the size limit for passive diffusion, clearly accumulated in nuclei of transfected cells, confirming an active mechanism of nuclear import (Fig. 1*B*). We also used the same protein backbone (GFP-GST) to analyze the localization of fragments of NOSIP. Surprisingly, proteins containing N-terminal fragments of NOSIP (amino acids 1–110, 75–102, 1–160, or 1–240), all of which contain the putative NLS, did not reach the same level of nuclear accumulation as the full-length protein or were even excluded from the nucleus (Fig. 1*B*).

GFP-GST-NOSIP^111−240^ and GFP-GST-NOSIP^111−301^, on the other hand, showed an equal distribution between the nucleus and the cytoplasm in many cells. Interestingly, NOSIP fragments containing amino acids 75 to 140, 75 to 180, or 75 to 200 (*i.e.*, fragments containing the putative NLS) did not lead to a clear nuclear accumulation, suggesting that nucleocytoplasmic transport of NOSIP is more complex than expected.

Next, we performed heterokaryon assays to analyze nuclear import and also nuclear export of NOSIP in more detail. In our experimental setup, HeLa cells are transfected with a protein of interest and then cocultured with mouse NIH3T3 cells, whose nuclear morphology is clearly distinct from that of HeLa cells. Upon fusion of cells as induced by polyethylene glycol, potential export of the protein of interest from HeLa to NIH3T3 cells can be analyzed. As a control, we used the reporter protein Rev^47−116^-GFP_2_-cNLS, which is exported *via* the CRM1 pathway and imported *via* importin α/β. As shown in Figure 1*C*, Rev^47−116^-GFP_2_-cNLS was observed in NIH3T3 nuclei upon cell fusion, indicating nuclear export from the human HeLa nucleus followed by import into the mouse nucleus. Shuttling was inhibited by LMB, confirming CRM1 as the relevant export receptor for Rev^47−116^-GFP_2_-cNLS. For NOSIP, we used two versions for the heterokaryon assay: NOSIP-HA, which has a similar size as the endogenous protein (34 versus 35 kDa) and the much larger GFP-GST-NOSIP (87 kDa). As expected, NOSIP-HA was found to shuttle from HeLa nuclei to NIH3T3 nuclei. In contrast to Rev^47−116^-GFP_2_-cNLS, however, shuttling was not inhibited by LMB, supporting the previous notion that CRM1 is not involved in nuclear export of NOSIP. Accordingly, GFP-GST-NOSIP did not shuttle in our assay, suggesting that there is no active export of NOSIP, neither CRM1 dependent nor CRM1 independent. To further analyze nucleocytoplasmic shuttling of NOSIP, we took advantage of an approach where nuclear import and export of a reporter protein can be controlled. In this assay, the protein of interest is linked to a fusion protein comprising GFP and a portion of the glucocorticoid receptor (GR) (21). As a control, we used the full-length HIV-1 Rev protein, which contains both nuclear import and nuclear export sequences. As shown in Figure 1*D*, Rev-GR-GFP was imported into the nuclei of transfected cells upon addition of the hormone analogue dexamethasone. After medium exchange (*i.e.*, washout of the hormone), Rev-GR-GFP partially returned to the cytoplasm. Similarly, GR_2_-GFP-NOSIP clearly accumulated in the nucleus upon addition of dexamethasone. In contrast to Rev-GR-GFP, however, it was largely retained in the nucleus after a 2-h export reaction. Together, these results show that NOSIP can be actively imported into the nucleus. Its nuclear export, by contrast, seems to depend on passive diffusion.

### Interaction of NOSIP with NTRs

**Figure 2:**
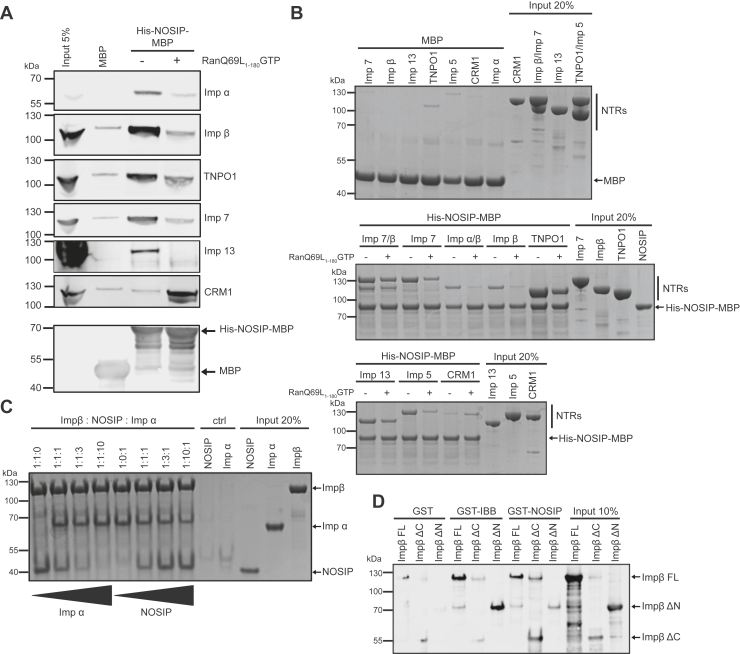
**NOSIP directly interacts with several nuclear transport receptors (NTRs) in a RanGTP-dependent manner.***A*, His-NOSIP-MBP or MBP immobilized on MBP-selector beads was incubated with HeLa cytosol in the presence or absence of RanQ69L_1–180_ loaded with GTP, as indicated. Bound proteins were analyzed by SDS-PAGE, followed by Western blotting using antibodies against Imp α, Imp β, TNPO1, Imp 7, Imp 13, CRM1, and MBP, as indicated. *B*, His-NOSIP-MBP or MBP (*top panel*) immobilized on amylose beads was incubated with His-tagged NTRs (Imp α, Imp β, Imp 7, Imp 13, TNPO1, CRM1, Imp 5) in the presence or absence of RanQ69L_1–180_ loaded with GTP, as indicated. Bound proteins were analyzed by SDS-PAGE, followed by Coomassie staining. *C*, His-S-importin β was immobilized on S-protein beads and incubated with His-NOISP or His-importin α at different molar ratios as indicated. For control (ctrl), S-protein beads alone were used and incubated with His-NOISP or His-imp α. Bound proteins were analyzed by SDS-PAGE, followed by Coomassie staining. *D*, GST-IBB (importin β-binding domain of importin α), GST-NOSIP, or GST were immobilized on glutathione Sepharose beads and incubated with His-S-tagged versions of full-length importin β (Imp β FL) or C-terminal (ΔN) or N-terminal (ΔC) fragments. Bound proteins were analyzed by SDS-PAGE, followed by Western blotting using an antibody against importin β.

NOSIP was previously shown to interact with importin α in pull-down experiments using the import receptor GST-importin α as the immobilized protein (14). Potential binding of alternative NTRs was not addressed in this study. We therefore took a more systematic approach and immobilized NOSIP *via* an MBP-(maltose binding protein) tag on beads to investigate its interaction with different transport receptors from a cellular lysate. To control the specificity of the interactions, reactions were performed in the presence or absence of RanQ69L-GTP, a mutant version of Ran that is insensitive to the GTPase-promoting activity of RanGAP (22). Indeed, importin α bound to immobilized His-NOSIP-MBP and binding was reduced in the presence of RanGTP (Fig. 2*A*). In addition to importin α, most tested import receptors, *i.e.*, importin β, TNPO1, importin 7, and importin 13, were detected in the bound fraction in a RanGTP-dependent manner. Importin 9 and importin 11 did not bind under our experimental conditions (data not shown). Surprisingly, also the export receptor CRM1 was found to interact specifically with His-NOSIP-MBP. In contrast to the import receptors, strong binding of CRM1 was only observed in the presence of RanGTP, *i.e.*, in an exportin-typical manner. No significant binding of importins or exportins was observed when MBP instead of His-NOSIP-MBP was immobilized on beads. We also investigated binding of purified NTRs to immobilized His-NOSIP-MBP. Similar to the results obtained with cytosol as a source of transport factors (Fig. 2*A*), all tested NTRs interacted with His-NOSIP-MBP in a RanGTP-dependent manner (Fig. 2*B*). Importantly, importin β was found to bind directly to NOSIP, *i.e.*, independently of importin α. The latter did not further promote importin β binding and was hardly detected in the bound fraction. Furthermore, importin 7 alone bound to His-NOSIP-MBP. When importin β and importin 7 were incubated together, both NTRs interacted with immobilized NOSIP. Compared with protein levels in the input, strongest binding to NOSIP was observed for the import receptor TNPO1 in this qualitative assay, which was only partially reduced in the presence of RanGTP. For CRM1, by contrast, RanGTP slightly promoted binding to His-NOSIP-MBP. In control reactions, very little amounts of NTRs bound to immobilized MBP (Fig. 2*B*, top). We next investigated if NOSIP competes with importin α for the same binding site on importin β. His-S-importin β was immobilized *via* the S-protein-tag and incubated with NOSIP and increasing amounts of importin α or with a constant amount of importin α and increasing amounts of NOSIP. Importin α in a 10-fold molar excess clearly replaced NOSIP from importin β, whereas a 10-fold molar excess of NOSIP resulted in a partial replacement of importin α (Fig. 2*C*).

Competition of NOSIP and importin α for binding sites on importin β led to the assumption that NOSIP may bind to the C-terminal arch of importin β, similar to the importin β–binding domain (IBB domain) of importin α (23). To test this hypothesis, we analyzed binding of full-length importin β (FL), importin β ΔC (aa 1–396, lacking the IBB-binding site) and importin β ΔN (aa 304–876, containing the IBB-binding site) to immobilized GST-NOSIP or GST-IBB. As expected, FL-importin β and importin β ΔN bound to GST-IBB (Fig. 2*D*). Importin β ΔN bound only weakly to GST-NOSIP, in contrast to importin β ΔC and importin β FL (Fig. 2*D*), suggesting a different and perhaps overlapping binding site of NOSIP for importin β.

**Figure 3:**
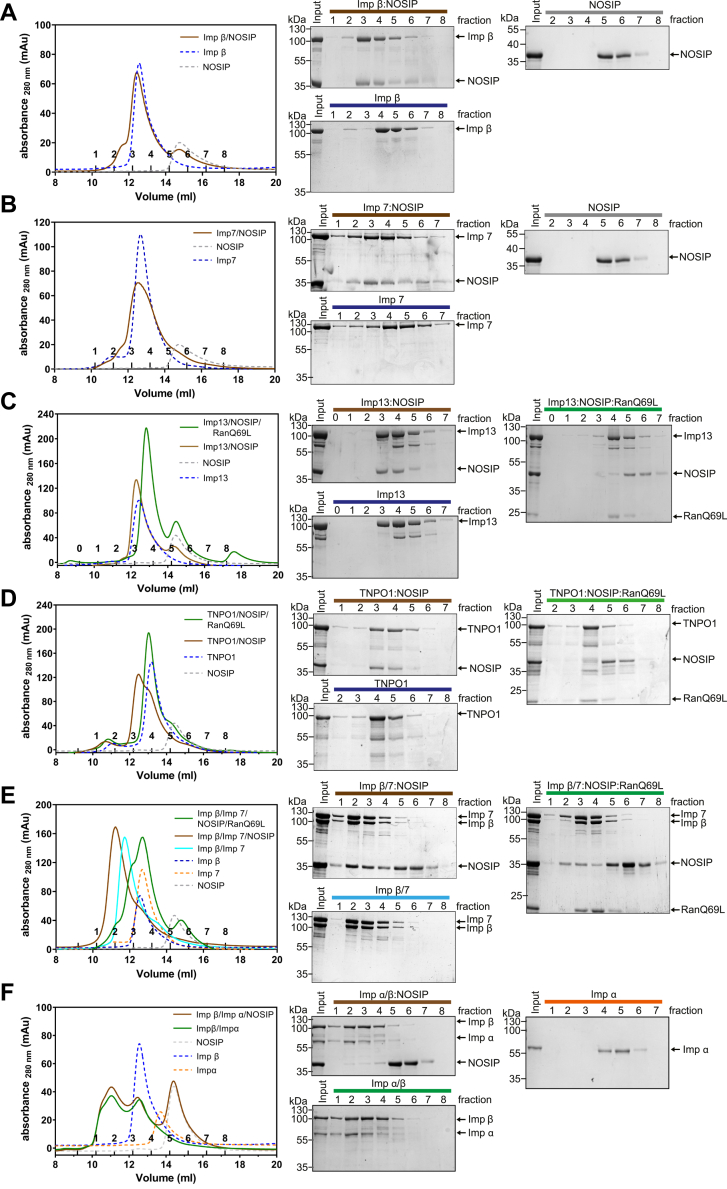
**Several nuclear transport receptors (NTRs) form stable complexes with NOSIP.** His-NOSIP was preincubated with His-tagged NTRs (Imp β, Imp 7, Imp 13, TNPO1, Imp β/7, and Imp β/α) as indicated at a ratio of 1:1 (*A–D*) or of 1:1:3 (NTR 1: NTR 2: NOSIP; *E–F*) for trimeric complexes. After size exclusion chromatography monitoring the absorbance at 280 nm, protein-containing fractions were analyzed by SDS-PAGE, followed by Coomassie staining. *A* and *B*, all samples in this experiment were analyzed together, and the two gels depicting NOSIP alone (*right*) are identical.

Next, we performed size exclusion chromatography for a more stringent analysis of the interaction of NOSIP with NTRs. For these experiments, His-NOSIP lacking the MBP-tag was used. First, single NTRs were preincubated with His-NOSIP and then subjected to gel filtration. Importin β, importin 7, importin 13, and TNPO1 formed stable complexes with NOSIP, which coeluted from the column in the same fractions (Fig. 3, *A*–*D*). For importin 13 (Fig. 3*C*) and TNPO1 (Fig. 3*D*) we also performed reactions in the presence of RanQ69L-GTP, which prevented the formation of stable complexes. We then addressed the possibility of formation of trimeric import complexes containing NOSIP, importin β and importin α, or importin β and importin 7, respectively. For the latter condition, we observed coelution of NOSIP, importin β, and importin 7 in the same fractions, suggesting the formation of a trimeric complex (Fig. 3*E*). In the presence of importin α, by contrast, the two import receptors coeluted in the same fractions, whereas NOSIP eluted much later from the column (Fig. 3*F*). Clearly, its elution peak was not shifted compared with a reaction with NOSIP alone. These results suggest that several NTRs can serve as import receptors for NOSIP. The protein does not, however, bind to the importin α/β dimer under our experimental conditions.

**Figure 4:**
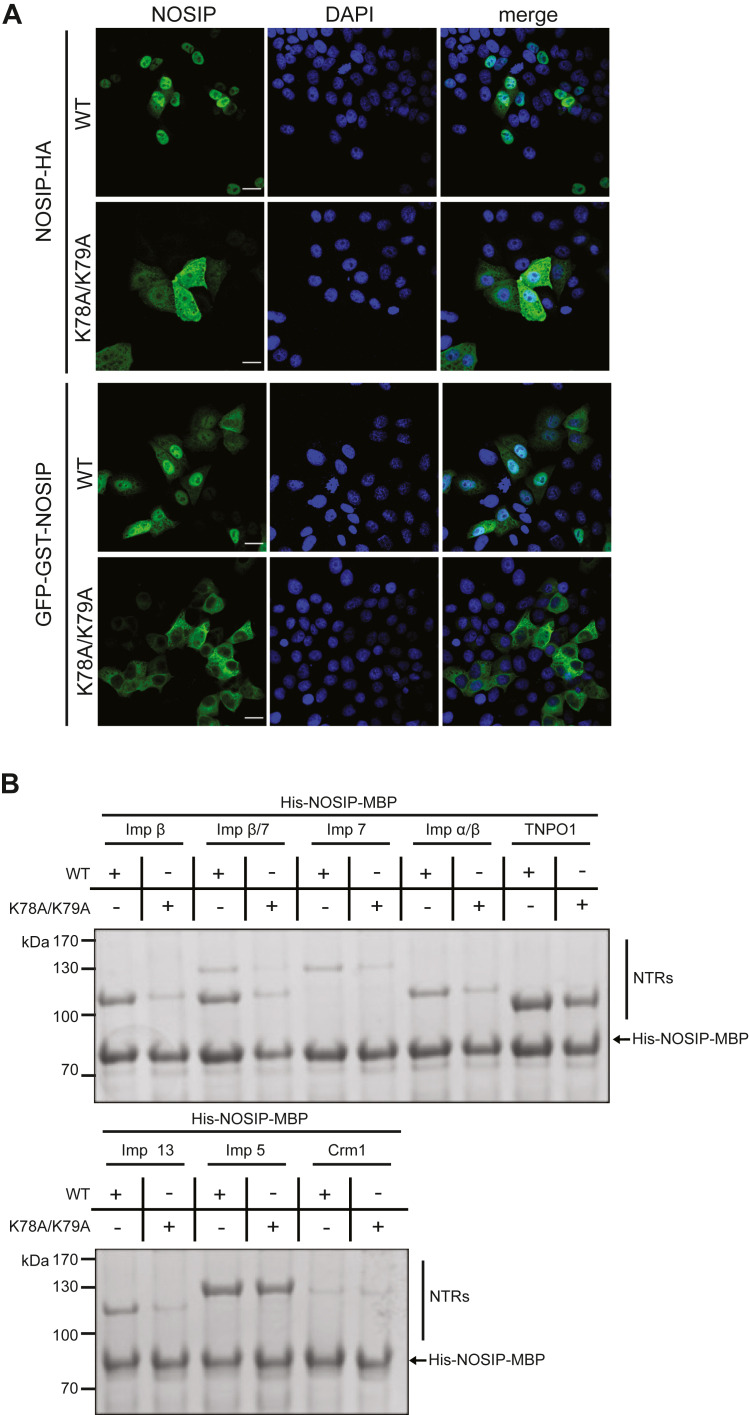
**Mutation of NOSIP’s nuclear localization signal abolishes the interaction with nuclear transport receptors (NTRs).***A*, HeLa cells were transfected with constructs coding for GFP-GST- or HA-tagged versions of wildtype (WT) or mutant (K78AK79A) NOSIP. NOSIP (*green*) was visualized *via* the GFP-tag or by indirect immunofluorescence using anti-HA antibodies. Nuclei were stained with DAPI (*blue*). Cells were analyzed by confocal microscopy. The scale bars represent 20 μm. *B*, Wildtype (WT) His-NOSIP-MBP or His-NOSIP-MBP K78AK79A was immobilized on amylose beads and incubated with His-tagged NTRs (Imp α, Imp β, Imp 7, Imp 13, TNPO1, CRM1, or Imp 5, as indicated). Bound proteins were analyzed by SDS-PAGE, followed by Coomassie staining.

**Figure 5:**
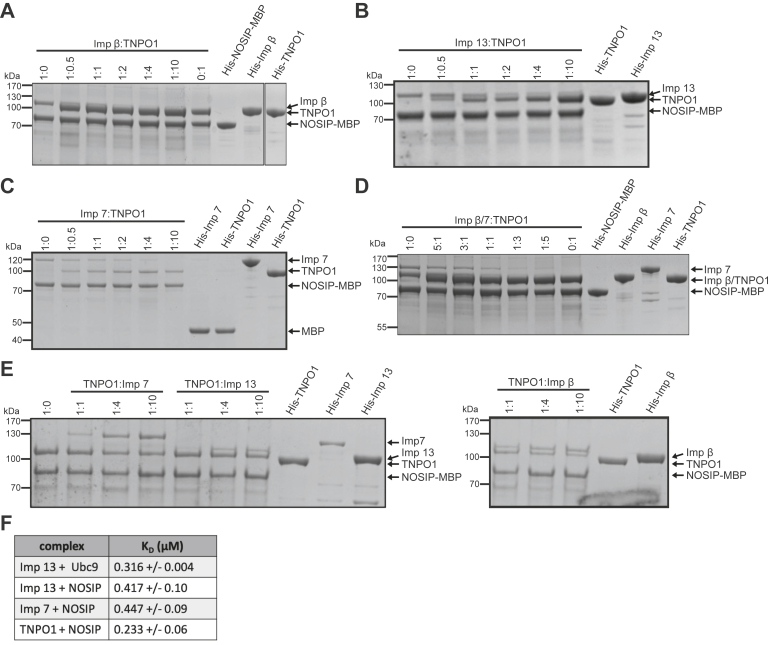
**Transportin has a high affinity for NOSIP.***A–D*, for competition among nuclear transport receptors, His-NOSIP-MBP was immobilized on amylose beads and incubated with His-tagged nuclear transport receptors. Importin β (*A*), importin 13 (*B*), importin 7 (*C*), and importin β plus importin 7 (*D*) were used at a molar ratio of 1:1 with respect to His-NOSIP-MBP and incubated with increasing molar ratios of His-TNPO1, as indicated. *C*, MBP was immobilized as a control and incubated with importin 7 or importin β, as indicated. *E*, His-NOSIP-MBP was immobilized as above and incubated with TNPO1 at a molar ratio of 1:1 and increasing amounts of importin 7, importin 13, or importin β, as indicated. *A–E*, bound proteins were analyzed by SDS-PAGE, followed by Coomassie staining. *F*, K_D_-values were determined using differential scanning fluorimetry (see Fig. S1 for details).

**Figure 6:**
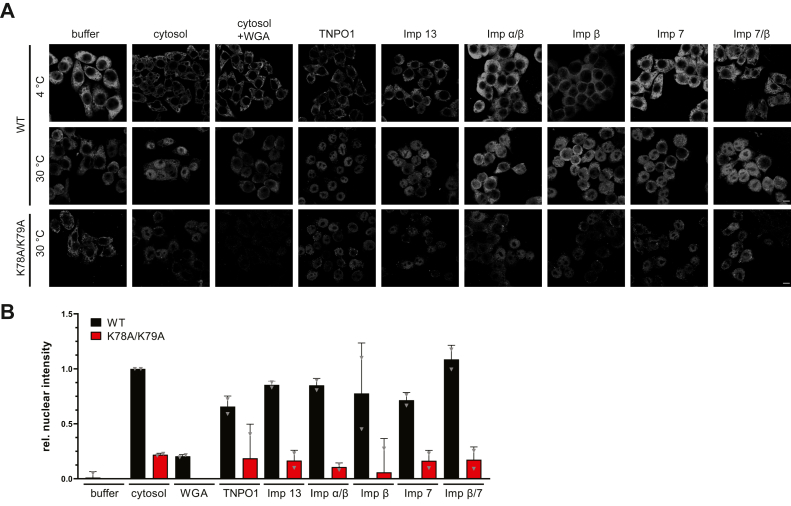
**NOSIP is imported by various nuclear transport receptors *in vitro*.***A*, HeLa cells were permeabilized with digitonin and incubated with His-NOSIP-MBP WT or K78AK79A, Ran, ATP-regenerating system, and either cytosol or His-tagged nuclear transport receptors (TNPO1, Imp 13, Imp α/β, Imp β, Imp 7, Imp β/7, or His-Imp β) at 4 °C or 30 °C as indicated. His-NOSIP-MBP was visualized by indirect immunofluorescence using anti-MBP antibodies. Cells were analyzed by confocal microscopy. The scale bars represent 10 μm. *B*, quantification of the results presented in *A*. The mean nuclear fluorescence intensities of 4 °C reactions were subtracted from the respective 37 °C values and normalized to the value obtained for the reaction containing cytosol, which was arbitrarily set to 1.

Our findings described so far raised the question of whether the NLS that had previously been identified for NOSIP (14) (Fig. 1*A*) mediates binding to all different NTRs. Point mutations in the NLS (K78A/K79A) resulted in reduced nuclear localization of NOSIP-HA compared with the wildtype protein (Fig. 4*A*), similar to previous results for a myc-tagged version (14). We also introduced the mutations into the large construct coding for GFP-GST-NOSIP. In this context, they completely abolished nuclear localization of the fusion protein, further demonstrating the functionality of the NLS. Finally, we analyzed binding of wildtype NOSIP and NOSIP K78A/K79A, both immobilized as His-MBP-tagged proteins, to individual NTRs. As shown in Figure 4*B*, the mutations clearly reduced binding to importin β (alone or in reactions with importin α or importin 7), to importin 7, importin 13, and TNPO1. Only a small reduction was observed for importin 5 and no binding was seen for CRM1, as expected. Together, our binding experiments suggest that multiple NTRs could function as import receptors and that they use the same region in NOSIP as NLS. NTRs, however, may differ in their affinities for NOSIP. We therefore performed competition experiments with immobilized NOSIP. First, we added increasing concentrations of TNPO1 to reactions containing a fixed amount of importin β, importin 13, or importin 7. A 2-fold molar excess of TNPO1 could largely prevent binding of importin β (Fig. 5*A*), importin 13 (Fig. 5*B*), and importin 7 (Fig. 5*C*) to NOSIP. Preferential binding of TNPO1 was also observed when the reaction contained importin β together with importin 7 (Fig. 5*D*). In reverse experiments, we kept the concentration of TNPO1 constant and included increasing amounts of importin 7, importin 13, or importin β in the reactions. Even at a 10-fold molar excess of these NTRs compared with TNPO1, the latter still interacted with immobilized NOSIP (Fig. 5*E*), suggesting that the affinity of TNPO1 for NOSIP is comparatively high. Next, we measured affinities of NOSIP for different NTRs in solution, using differential scanning fluorometry, a method that has previously been used to determine the affinity between importin 13 and its import cargo Ubc9 (24). Our measured K_D_-value for the importin 13-Ubc9 interaction (0.316 ± 0.04 μM; Fig. 5*F*) was very similar to the published value (0.37 μM) using the same method. All tested NTRs bound NOSIP with affinities in the submicromolar range, where TNPO1 showed the highest affinity (0.233 ± 0.06 μM), followed by importin 13 (0.417 ± 0.1 μM) and importin 7 (0.447 ± 0.09 μM). Together, these results suggested that several NTRs are theoretically able to function as import receptors for NOSIP and that TNPO1 is a preferred binding partner. We directly tested this hypothesis and performed nuclear import assays in permeabilized cells, using purified proteins as import cargos and transport factors, respectively. As shown in Figure 6, *A* and *B*, wildtype His-NOSIP-MBP was readily imported into the nucleus in the presence of cytosol. Wheat germ agglutinin, a lectin that inhibits active nuclear import (25, 26), strongly reduced the nuclear fluorescence, confirming the specificity of the import reaction. Import of the NOSIP mutant K78A/K79A was also reduced compared with the wildtype protein, as expected from our results described above. Very similar observations were made when purified NTRs (TNPO1, importin 13, importin α/β, importin β, importin 7, importin β/7) were used instead of cytosol, further strengthening the notion that NOSIP can be imported by more than one NTR. For all NTRs, the identified NLS seems to be critical.

### Transportin 1 is a major import receptor of NOSIP in HeLa cells

**Figure 7:**
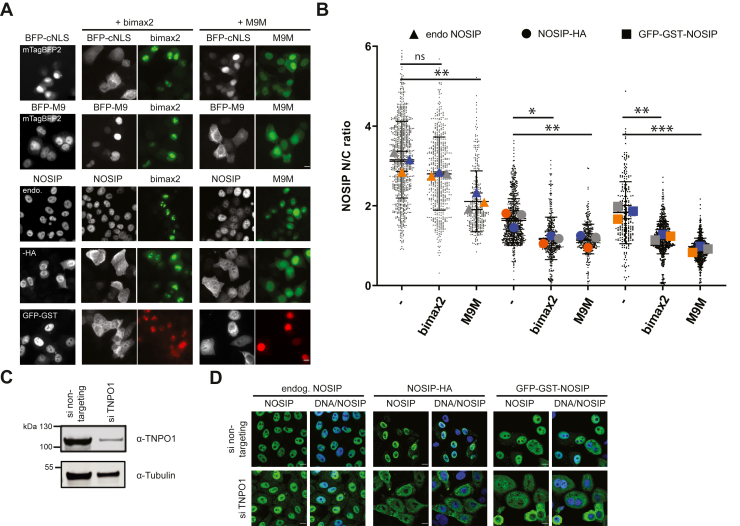
**Transportin 1 is a major import receptor for NOSIP.***A*, HeLa cells were cotransfected with plasmids coding for HA- or GFP-GST-tagged versions of NOSIP and GFP- or RFP-tagged versions of the inhibitory peptide sequences M9M or bimax2, as indicated. As controls, cells were transfected with control constructs coding for the shuttling proteins BFP-cNLS (NES-mTagBFP_2_-cNLS) or BFP-M9 (NES-mTagBFP_2_-M9) instead of NOSIP constructs. NOSIP or controls are depicted in *gray*; M9M/bimax2 are depicted in the color of their respective fluorescence tag. Images were acquired by confocal microscopy. The scale bars represent 10 μm. *B*, quantification of the results in (*A*). N/C ratios of NOSIP and cNLS/M9 reporter proteins were analyzed using cell profiler software and are presented as mean values ± SEM of three independent experiments (*gray*, *blue*, and *orange* symbols) with a total of 500 to 1000 cells per condition. *p*-values, ns: >0.05, ∗: ≤0.05, ∗∗: ≤0.01, ∗∗∗: ≤0.001. *C*, siRNA mediated knockdown of transportin. HeLa cells were transfected with siRNAs against TNPO1 (si TNPO1) or with nontargeting siRNAs, as indicated. Cell lysates were analyzed by Western blotting, detecting TNPO1 or tubulin as loading control. *D*, cells were cotransfected with plasmids coding for HA- or GFP-GST-tagged NOSIP and with control siRNAs or siRNAS against TNPO1 as indicated. Endogenous (endog.) or overexpressed NOSIP (*green*) was visualized by indirect immunofluorescence with anti-HA or anti-NOSIP antibodies or directly *via* the GFP-tag. Nuclei were stained with DAPI (*blue*) and cells were analyzed by confocal microscopy. The scale bars represent 10 μm.

**Figure 8:**
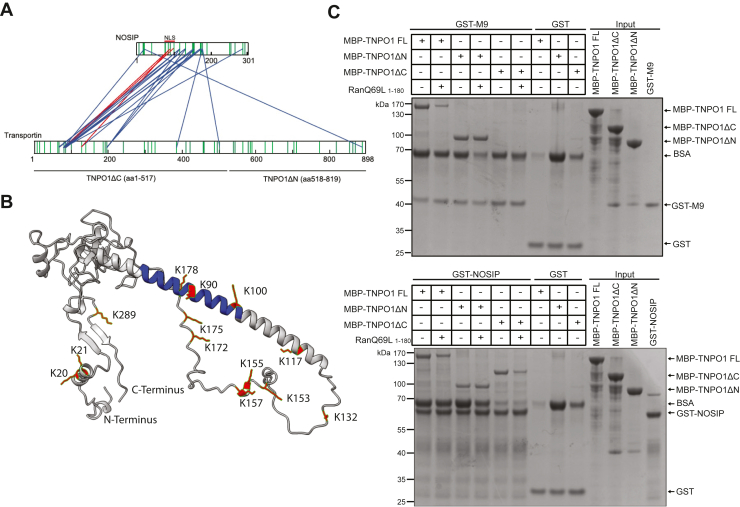
**Unconventional binding of NOSIP to transportin 1.***A*, the His-NOSIP/His-TNPO1 complex was purified by size exclusion chromatography, treated with BS3, and cross-linked peptides were analyzed by mass spectrometry. Identified cross-links are depicted with *blue lines*. *Red lines* indicate cross-links between TNPO1 and the nuclear localization signal of NOSIP. *Green bars*, lysins in NOSIP and transportin. *B*, cross-linked lysines mapped on the predicted structure of NOSIP. The structure was obtained using AlphaFold (29) and modified with respect to coloring. *Blue*, identified nuclear localization signal (aa 78–101) of NOSIP; red, all identified lysines after BS3 cross-linking are depicted as sticks. Images were created using ChimeraX 1.3 software (66). *C*, GST-M9 (*top*) or GST-NOSIP (*bottom*) was immobilized on glutathione Sepharose beads and incubated with MBP-tagged versions of full-length TNPO1 (TNPO1 FL) or of C-terminal (ΔN) or N-terminal (ΔC) fragments in the presence or absence of RanQ69L_1–180_ that had been loaded with GTP, as indicated. Bound proteins were analyzed by SDS-PAGE, followed by Coomassie staining.

To further address the question of which NTR serves as the physiological import receptor for NOSIP in living cells, we performed transfection experiments with established inhibitors of two prominent import pathways. Bimax2 is a short peptide with a very high affinity for importin α (27). Fused to a fluorescent reporter protein, it inhibits importin α/β-dependent, but not, for example, transportin-dependent import (27). M9M, on the other hand, is a peptide with high affinity for TNPO1 that inhibits transportin-dependent nuclear import but not other transport pathways (28). To control the specificity of these inhibitors, we used BFP-tagged reporter proteins with a classic NLS (cNLS) or an M9-sequence, which mediate importin α/β or transportin-dependent import, respectively. Indeed, GFP-bimax2 inhibited nuclear import of NES-BFP-cNLS but not of NES-BFP-M9, whereas reduced import of NES-BFP-M9 but not of NES-BFP-cNLS was observed in cells expressing GFP-M9M (Fig. 7*A*). We then examined the subcellular localization of endogenous NOSIP, of NOSIP-HA, and of GFP-GST-NOSIP in cells expressing the two inhibitory proteins. A clear shift of endogenous NOSIP toward the cytoplasm was only observed in cells expressing GFP-M9M (Fig. 7, *A* and *B*), suggesting that TNPO1 functions as a major import factor. Nuclear import of the overexpressed versions of NOSIP, NOSIP-HA, and GFP-GST-NOSIP, on the other hand, was inhibited by both bimax2 and M9M, fused to GFP or RFP, respectively (Fig. 7, *A* and *B*). Next, we performed siRNA depletion experiments to reduce the cellular concentration of TNPO1 (Fig. 7*C*). As shown in Figure 7*D*, this depletion resulted in a minor shift of endogenous NOSIP toward the cytoplasm. This was not observed upon depletion of either importin β or importin 13 (data not shown). As seen for the competing import inhibitors, inhibition of nuclear import of overexpressed NOSIP upon depletion of TNPO1 was more pronounced than that of the endogenous protein. Two independent siRNAs against TNPO1 were less efficient in depleting the transport receptor. Nevertheless, reduced nuclear import of overexpressed NOSIP-fusion proteins was observed in experiments using these siRNAs (Fig. S2). Together, these results suggest that TNPO1 is a major nuclear import receptor for NOSIP in living cells. We therefore decided to analyze the interaction of NOSIP with TNPO1 in more detail and performed cross-linking experiments with the purified NOSIP–TNPO1 complex using BS3 as a lysine-reactive reagent. Cross-linking sites were identified by mass spectrometry, revealing 24 intermolecular cross-links between NOSIP and TNPO1 (for identified cross-links see Table S1). For NOSIP, most cross-links were found to lysine residues in the region of the protein comprising amino acids 85 to 180 of NOSIP, including to K90 and K100 of NLS (Fig. 8, *A* and *B*). We used an AlphaFold prediction of the NOSIP structure (29) to map the identified sites of interaction. They are mainly distributed along an extended alpha-helix, which is followed by an intrinsically disordered region (Fig. 8*B*). The predicted folded part of NOSIP, seems to be not involved in binding to TNPO1.

For TNPO1, the vast majority of cross-links was found within the first 200 amino acids of the protein. The interaction of TNPO1 with a classic M9-containing cargo, on the other hand, is known to involve rather the C-terminal portion of the import receptor (30, 31). We therefore compared binding of full-length TNPO1 and N- and C-terminal deletion mutants to immobilized GST-M9 and GST-NOSIP. As shown before (32), full-length TNPO1 and TNPO1 ΔN (aa 518–890) bound to GST-M9. For the full-length protein, binding was reduced in the presence of RanGTP (Fig. 8*C*, top). TNPO1 ΔC (aa 1–517), on the other hand, did not bind to GST-M9. This was in stark contrast to the results for immobilized GST-NOSIP (Fig. 8*C*, bottom), which interacted with all versions of TNPO1, including TNPO1 ΔC. These results point to an interaction mode of NOSIP with TNPO1 that is distinct from that of a classic, M9-containing import substrate.

## Discussion

### Nuclear shuttling of NOSIP

At elevated concentrations, NO was shown to have antiproliferative effects leading to a cell-cycle arrest at the G1/S transition. Lower NO levels, by contrast, favor cell-cycle progression and proliferation (33). The interaction of eNOS, a major cytoplasmic NO-producing enzyme, with NOSIP may therefore help to regulate key steps during the cell cycle. As an inhibitor of eNOS activity, nuclear import of NOSIP would sequester it away from the cytoplasmic enzyme, keeping NO concentrations at basal levels. Inhibition of eNOS would only become relevant under conditions of reduced nuclear import or enhanced nuclear export of NOSIP, as observed in the G2 phase of the cell cycle (14). Interaction of NOSIP with eNOS may thus reduce NO production to favor cell-cycle progression.

In this study, we therefore investigated nuclear import and export of NOSIP in more detail. Several lines of evidence showed that nuclear export of NOSIP does not occur in an active manner: first, our heterokaryon assays confirmed that NOSIP-HA is a nuclear shuttling protein (14); its nuclear export, however, was not inhibited by LMB, ruling out CRM1 as a potential export factor under our experimental conditions. Second, the much larger protein, GFP-GST-NOSIP, did not shuttle in this assay, suggesting that there is no active export of this protein at all (Fig. 1*C*). We cannot completely rule out the possibility, however, that the large tag at the N terminus of NOSIP interferes with the interaction of NOSIP with CRM1 or another, as yet unidentified, nuclear export receptor. Third, in a transport assay, where the subcellular localization of reporter proteins can be controlled by the addition of dexamethasone to the cells and the subsequent withdrawal of the drug, no significant export of GR_2_-GFP-NOSIP could be observed (Fig. 1*D*). We conclude that NOSIP is actively imported into the nucleus but leaves the nucleus by passive diffusion. Our observation of RanGTP-dependent binding of NOSIP to CRM1 (Fig. 2, *A* and *B*) may point to a complex and as yet unidentified role of CRM1 in NOSIP shuttling under very specific conditions, *e.g.*, during cellular stress or at the G2 stage of the cell cycle, where NOSIP accumulates in the cytoplasm (14). A change in NOSIP nuclear transport could result from posttranslational modifications, *e.g.*, phosphorylation. Indeed, NOSIP was shown to be phosphorylated during mitosis at S36 and S138 (34).

### NOSIP interacts with several nuclear transport receptors

NOSIP was suggested to bind to importin α and to be transported through the canonical importin α/β pathway (14). In this study, GST-importin α was incubated with a lysate from HeLa cells overexpressing NOSIP and NOSIP binding could be detected. In-depth binding studies using purified proteins, however, were not performed by the authors. We also observed binding of NOSIP to importin α from a HeLa cell lysate (Fig. 2*A*). However, we could not detect direct importin α binding to NOSIP using purified proteins. Instead, we observed an importin α−independent binding of importin β (Fig. 2*B*). Accordingly, functional NOSIP-importin α/β-trimers could not be observed in gel filtration experiments (Fig. 3). Competition experiments further suggested that importin α and NOSIP compete for similar binding sites on importin β (Fig. 2*C*). Nuclear transport assays using digitonin-permeabilized cells supported the notion that importin β alone can promote nuclear import of NOSIP, as the addition of importin α together with importin β did not lead to enhanced import of NOSIP compared with a reaction with importin β alone (Fig. 6). Together, these results suggest that importin α is not a relevant import factor for NOSIP. This is further supported by the notion that bimax2, an inhibitor of importin α, had a rather small effect on the subcellular localization of endogenous NOSIP, compared with M9M, an inhibitor of TNPO1 (Fig. 7, *A* and *B*).

Recently, we identified NOSIP as an interaction partner of the alternative transport receptor importin 13 (18). A systematic analysis of potential interaction partners revealed that not only importin 13 but also several other NTRs, namely, importin β, importin 5, importin 7, and TNPO1 (Fig. 2), bind NOSIP. Interaction was specific, as it was reduced in the presence of RanGTP (Fig. 2*B*) or with an NLS mutant of NOSIP (Fig. 4*B*). Furthermore, our *in vitro* transport assays using permeabilized cells showed that NOSIP is efficiently imported by all of the tested NTRs (Fig. 6).

### Transportin 1 is the major nuclear transport receptor for NOSIP

Several lines of evidence suggest that TNPO1 is a major import receptor for NOSIP in living cells, at least in HeLa cells. First, inhibition of the transportin-dependent import pathway with the competitive inhibitor M9M resulted in reduced nuclear accumulation of endogenous NOSIP. The observed effect was much stronger than that for a competitive inhibitor of the importin α/β−pathway, bimax2 (Fig. 7, *A* and *B*). The latter only had strong effects on nuclear import of exogenous, overexpressed NOSIP, *i.e.*, under conditions where the physiological NTR might become rate limiting. Second, depletion of TNPO1 with specific siRNAs slightly reduced nuclear accumulation of endogenous NOSIP. Again, this effect was more pronounced for exogenous NOSIP (NOSIP-HA or GFP-GST-NOSIP). These data are in line with our *in vitro* nuclear import data, where transportin, as other NTRs, was able to support import of NOSIP into the nucleus (Fig. 6). Furthermore, our biochemical data showed robust and specific binding of NOSIP to TNPO1 (Figs. 2 and 4) and TNPO1 showed the highest affinity of the tested NTRs for NOSIP (Fig. 5). The concentration of TNPO1 in HeLa cells is in the range of 1 μM, similar to that of importin β and importin 7, but much higher than that of importin 13 (35). Together, our data suggest that TNPO1 is a major import receptor and that alternative NTRs can support nuclear import of NOSIP under conditions of high cargo or low TNPO1 concentrations, as they may occur in certain differentiated cells. Thus, it remains to be investigated how NTR-dependent nuclear transport of NOSIP might affect the activity of eNOS and NO signaling, *e.g.*, in endothelial cells.

### NOSIP-transportin: an unusual mode of interaction

The best-known interaction mode of TNPO1 with its cargoes is through a signal sequence termed PY-NLS (M9-sequence), first identified in hnRNPA1 (36) and later found in other proteins like hnRNP M, hnRNP D, FUS, TAP, and HCC1 (31). The PY-NLS, which consists of a hydrophobic or basic N-terminal motif followed by a conserved basic residue and a PY motif (R/H/K-X_2−5_-PY), binds to the C-terminal arch of TNPO1 (30). Furthermore, TNPO1 is known to import proteins lacking a typical PY-NLS, like histones, ribosomal proteins, ADAR1, and CIRBP (31). These non-PY-NLS interact through various ways with transportin: CIRBP (cold-inducible RNA-binding protein) nuclear import and interaction with TNPO1 is mediated by an RG/RGG-rich region (37); histone H3 binds similar to a PY-NLS, without occupying the PY motif–binding site of TNPO1 (38); ADAR1 (adenosine deaminase acting on RNA 1) contains two NLS modules, which are separated from each other and form a functional NLS upon correct folding called “bimodular NLS” (39).

NOSIP does not contain a typical PY-NLS or a similar sequence lacking the PY motif or an RG/RGG-rich region. Hence, NOSIP belongs to the group of non-PY-NLS TNPO1 cargoes.

Our cross-linking approach revealed an unusual interaction mode between NOSIP and transportin. NOSIP interacts mainly with the N-terminal part (aa 1–200) of TNPO1 and only barely with the C-terminal part, which could be confirmed by binding studies using respective N- or C-terminal fragments of TNPO1 (Fig. 8). This was surprising, since almost all of the reported cargo interactions (PY-NLSs or non-PY-NLSs) involve the C-terminal arch (HEAT repeats 8–20) of TNPO1 (30, 31). The N-terminal arch (HEAT repeats 1–13), where NOSIP binds to transportin, is the binding site for Ran-GTP, which is conserved throughout the karyopherin-β family (40). To the best of our knowledge, there are only two additional TNPO1 cargoes known to bind to the N-terminal region of transportin, the transcription factor c-Fos (32) and the RNA-binding protein Rev of the human immunodeficiency virus type 1 (41).

The region of NOSIP involved in binding to TNPO1 could be mapped to NOSIP^85−180^, including a part of the identified NLS NOSIP^78−101^ (Fig. 8*A*), confirming the importance of the NOSIP-NLS for the interaction with TNPO1. This finding is further supported by the reduced binding of all tested NTRs to a NOSIP-NLS mutant, where two lysine residues were exchanged for alanines (Fig. 4*B*). With the assumption that this mutation does not affect the overall structure of NOSIP, these data suggest that this region is important for all NTRS. Fusing the identified NLS to GFP-GST and extending the NLS C-terminally to NOSIP^200^ did not result in a predominant nuclear localization as seen for the full-length protein (Fig. 1*B*). Hence, our data point to a more complex nuclear import of NOSIP, where NTRs rather bind to larger protein domains instead of linear peptide sequences, as it is known for importin 13 cargoes (24).

In summary, our data show that NOSIP can interact with and get imported by multiple NTRs, with TNPO1 being the major NTR, at least in HeLa cells. Further, our data suggest an unusual binding mode of NOSIP to TNPO1. In contrast to most known TNPO1 cargoes, which bind *via* a PY-NLS or non-PY-NLS to the C-terminal arch, NOSIP binds to the N-terminal arch of transportin, *i.e.*, the Ran-binding region. This may directly affect the dissociation of an import complex in the nucleus with its high concentration of RanGTP.

## Experimental procedures

### Plasmids and molecular cloning

Plasmids pEGFP-GST-NOSIP, pcDNA3.1(+)-NOSIP-HA and pMal-His-NOSIP-MBP (18), pMal-c2-TNPO1 (FL [aa 1–890],ΔC [aa 1–517], ΔN [aa 518–890]), pTYB2-S-His-importin β (ΔC [aa 1–396], ΔN [aa304–876]), pGEX-KG-GST-IBB and pGEX-4T1-GST-M9 (32), RanWT (42), pQE80-His-importin 13 (19), pQE80-RanQ69L (1–180) (43), pQE80-His-importin 7 (*Xenopus laevis*, obtained from R. Ficner (44), pQE32-His-TNPO1 (45), pQE60-His-CRM1 (46), pRSETb-His-importin α (47), pET30a-His-importin β (48), pXGmLnt-Rev-GR-GFP (21), pEGFP-c1-GFP-M9M and pEGFP-c1-GFP-Bimax2 (49), pEGFP-c1-Rev(47–116)-GFP_2_-cNLS (50) and pcDNA3-NES-mTagBFP_2_-cNLS (51)) were described before.

The plasmid pEGFP-c1-GR(511–795)_2_-GFP_2_-MCS was generated by inserting two copies of the hormone-responsive fragment of rat glucocorticoid receptor *via* NotI and XhoI and XhoI and BcuI in front of GFP. A second GFP was inserted *via* BglII and Eco32I.

NOSIP fragments (aa 1–110, 1–160, 1–240, 111–240, 111–301, 75–102, 75–140, 75–180, 75–200) were amplified by PCR using pcDNA3.1(+)-NOSIP-HA as template and cloned *via* Gibson assembly into the pEGFP-GST vector. For tagged versions of NOSIP K78AK79A, site-directed mutagenesis was performed using oligonucleotides 5′-GTACATTCTGCACCAGGCGGCGGAGATTGCCCGGCAG and 5′-CTGCCGGGCAATCTCCGCCGCCTGGTGCAGAATGTAC using the corresponding wildtype plasmids as template. The coding sequence of NOSIP was amplified by PCR using NOSIP-HA as a template and cloned into pGEX-6P-1 *via* EcoRI and XhoI to generate GST-NOSIP, in pQLink-His *via* HindIII and NotI to obtain His-NOSIP and in pEGFP-C1-GR(511–795)_2-_GFP_2_-MCS using BglII and SalI and thereby replacing one GFP to obtain GR(511–795)_2_-GFP-NOSIP. pcDNA3-NES-mTagBFP_2_-M9 was generated by amplifying NES-mTagBFP_2_ by PCR using pcDNA3-NES-mTagBFP_2_-cNLS as template (51) and cloning it into pcDNA3 *via* EcoRI and EcoRV. The oligonucleotides 5′-AAAGATATCATGGGGAATTACAACAATCAGTCTTC and 5′-AAACTCGAGTCAATAGCCACCTTGGTTTCGTG were used to amplify the M9-sequence of hnRNPA1 and inserting it *via* EcoRV and XhoI into pcDNA3-NES-mTagBFP_2_ to obtain pcDNA3-NES-mTag-BFP_2_-M9. Plasmids coding for RFP-M9M and RFP-Bimax2 were obtained from Dr Dorothee Dormann (49).

### Protein expression and purification

His-NOSIP was expressed in JM109 cells grown in LB medium and induced at an *A*_600nm_ of 0.7 with 1 mM IPTG for 18 h at 18 °C. His-importin 13 was expressed in JM109 cells grown in 2× YT medium containing 30 mM K_2_HPO_4_ and 2% glycerol and induced at an *A*_600nm_ of 0.7 with 0.5 mM IPTG for 16 to 18 h at 18 °C. His-Imp13 and His-NOSIP were purified using buffer A (50 mM Tris pH 7.4; 500 mM NaCl; 10 mM Mg(OAc)_2_; 5% glycerol; 10 mM β-mercaptoethanol; 1 μg/ml each of leupeptin, pepstatin, and aprotinin; and 0.1 mM PMSF) over Ni-NTA agarose (Qiagen), followed by size exclusion chromatography over a HiLoad 16/600 Superdex 200 prepgrade column (Cytiva) equilibrated in size exclusion chromatography buffer (50 mM Tris pH 7.4, 200 mM NaCl, 2 mM DTT). His-NOSIP-MBP was expressed in JM109 cells grown in LB medium to an *A*_600nm_ of 0.7 and induced with 0.5 mM IPTG at 18 °C for 18 h. The protein was purified using Ni-NTA agarose beads (Qiagen), eluted with buffer A with 300 mM imidazole and further enriched over amylose resin (New England Biolabs) using buffer A containing 20 mM maltose for elution and dialyzed overnight at 4 °C against size exclusion chromatography buffer.

GST-NOSIP was expressed in JM109 cells grown in LB medium and induced with 0.5 mM IPTG at an *A*_600nm_ of 0.7 for 4 h at 30 °C. Bacteria were lysed in buffer B (50 mM Na_2_HPO_4_/NaH_2_PO_4_, pH 8.0, 300 mM NaCl, 5 mM MgCl_2_, 10% glycerol, 10 mM β-mercaptoethanol) containing 1% Triton-X 100; 1 μg/ml each of leupeptin, pepstatin, and aprotinin; and 0.1 mM PMSF and purified over glutathione Sepharose beads using buffer B containing protease inhibitors as above, followed by size exclusion chromatography with a HiLoad 26/600 Superdex 200 prepgrade column (Cytiva) equilibrated in 20 mM Tris pH 7.4, 100 mM NaCl, and 2 mM DTT.

MBP-TNPO1 FL/ΔC/ΔN was expressed in BL21DE3 cells grown in LB medium for 3 h at 37 °C and induced at an *A*_600_ of 0.7 with 0.5 mM IPTG. MBP proteins were purified using amylose resin (New England Biolabs) in MBP buffer (20 mM Tris pH 7.5; 200 mM NaCl; 5% glycerol; 2 mM DTT; 1 μg/ml each of leupeptin, pepstatin, and aprotinin; and 0.1 mM PMSF) and separated by a HiLoad 26/600 Superdex 200 prepgrade column using an Äkta system (Cytiva) in transport buffer TPB; (20 mM Hepes, pH 7.3, 110 mM KOAc, 2 mM Mg(OAc)_2_, 1 mM EGTA, 2 mM DTT).

His-TNPO1 (45), His-CRM1 (46, 52), His-Importin α (47), S-His-importin β (48), GST-M9 (32), RanQ69L1-180 (52), Ubc9 (53), and RanWT (42) were purified as described. RanQ69L (aa 1–180) was loaded with GTP as described (54). His-importin 5 was a gift from Achim Dickmanns.

### Binding assays

For binding assays with MBP proteins, 100 pmol His-NOSIP-MBP or MBP was immobilized on 10 μl amylose beads (NEB Biotechnologies, E8022L) equilibrated in transport buffer containing 10 mg/ml ovalbumin or bovine serum albumin (Sigma) for 1 h at 4 °C under gentle agitation. After three washing steps with TPB containing 10 mg/ml ovalbumin, the beads were incubated with 100 pmol of purified NTR in the presence or absence of 300 pmol RanQ69L_1−180_-GTP for 3 h at 4 °C under gentle agitation in a total volume of 500 μl. To remove unbound proteins, beads were washed three times with TPB. Bound proteins were eluted in 4× SDS-sample buffer at 95 °C and analyzed by SDS-PAGE (4–12% NuPAGE, Invitrogen).

Binding assays using GST proteins were performed as described above, using 100 pmol GST, GST-IBB, GST-M9, or GST-NOSIP, immobilized on 10 μl glutathione Sepharose beads (Cytiva) and 100 pmol of respective proteins, as indicated.

For competition assays, 100 pmol His-S-importin β was immobilized on 10 μl S-protein beads (Cytiva) and incubated with 100 pmol His-NOSIP and increasing amounts of His-importin α (0, 100, 300, 1000 pmol) or the other way around. For assays with NTRs competing for binding sites on NOSIP, 100 pmol His-NOSIP-MBP was immobilized on amylose beads and incubated with 100 pmol of one and increasing amounts of the other NTR.

For binding assays with HeLa-cytosol, 600 pmol His-NOSIP-MBP was immobilized on 62.5 μl MBP-selector beads (Nanotag Biotechnologies) in TPB containing 10 mg/ml bovine serum albumin (BSA, Sigma) and 1 μg/ml each of leupeptin, pepstatin, and aprotinin (Sigma). After washing the beads 3 times with buffer, immobilized His-NOSIP-MBP was incubated with 200 μl HeLa-cytosol (Ipracell; 14,3 mg/ml) in the presence or absence of 2000 pmol RanQ69L_1−180_-GTP for 6 h at 4 °C under gentle rotation, followed by four washing steps with TPB. Bound proteins were eluted in 4× SDS-sample buffer at 95 °C and analyzed by SDS-PAGE (4–12% NuPAGE, Invitrogen) followed by Western blotting using the Odyssey system (LI-COR).

### Size exclusion chromatography

For complex formation, His-NOSIP and purified NTRs were incubated in TPB at a molar ratio of 1:1 (except His-importin β:His-importin 7:His-NOSIP and His-importin α:His-importin β:NOSIP with a ratio of 1:1:3) in the presence or absence of a 3-fold molar excess of RanQ69L_1−180_-GTP for 1 to 2 h at 4 °C. Samples were cleared by centrifugation at 4 °C at 16,100*g* for 20 min and subjected to size exclusion chromatography using a Superdex S200 analytical increase 10/300 GL column (Cytiva) equilibrated in TPB. Fractions were analyzed by SDS-PAGE followed by Coomassie staining.

### Differential scanning fluorimetry

Differential scanning fluorometry was performed essentially as described (24). Briefly, His-tagged NTRs were used at a concentration of 0.5 μM in TPB (supplemented with 2 mM DTT) and His-NOSIP or Ubc9 were titrated in 18 steps to the NTR with concentrations ranging from 0.05 μM to 5 μM. All reactions were prepared in 96-well plates in total volumes of 40 μl, containing 5× SYPRO Orange (Thermo Fisher Scientific), added from a 5000× stock in dimethyl sulfoxide. Reactions were incubated for 30 min at room temperature (RT), and fluorescence was measured with a temperature gradient from 25 to 90 °C, using a qPCR system (M x 3000P , Stratagene) and the ROX filter set. The melting temperatures (Tm) were calculated by fitting the Boltzman equation to the individual curves. The Tm was then plotted against the concentration of NOSIP and K_D_ values were calculated using an exponential equation for fitting.

### Cell culture

HeLa P4 (55) and NIH3T3 cells were cultured in Dulbecco’s modified Eagle’s medium (DMEM) (Gibco Life Technologies) supplemented with 10% fetal bovine serum, 100 U/ml penicillin, 100 μg streptomycin, and 6 mM L-glutamine at 37 °C and 5% CO_2_.

For siRNA-mediated knockdown, HeLa cells were seeded in 24-well plates, transfected with 50 nM siRNA pool against TNPO1 (Santa Cruz Biotechnology Inc, sc-35737) or the independent siRNAs (GCAAAGAUGUACUCGUAAG [siRNA A] and GUAUAGAGAUGCAGCCUUA [siRNA B]; obtained from Sigma) or ON-Target plus nontargeting siRNA (Dharmacon, D-001810–01–50) using the calcium chloride method (56). After 24 h, cells were transfected again with siRNAs, together with 0.5 to 1 μg of plasmid DNA using the calcium chloride method. The next day, samples were either used for indirect immunofluorescence or for Western blotting using standard protocols.

### Transfection, immunofluorescence, and microscopy

A total of 50,000 HeLa cells were grown on coverslips in 24-well plates and transfected with 0.5 to 1 μg of plasmid DNA using the calcium chloride method (56). For indirect immunofluorescence, cells were washed twice with PBS for 3 min, followed by fixation with 3.7% formaldehyde in PBS for 10 min at RT and permeabilization with 0.5% Triton X-100 in PBS for 5 min. After washing twice with PBS, coverslips were blocked with 3% BSA in PBS for 10 min and incubated with primary antibodies diluted in 3% BSA in PBS for 1 h at RT in a dark humidity chamber. After washing, cells were incubated with Alexa Fluor secondary antibodies for 1 h at RT in the dark, washed twice with PBS for 3 min, mounted in Mowiol containing DAPI (except for cells transfected with pcDNA3-NES-mTagBFP2 constructs) and analyzed with an LSM 510-META confocal laser scanning microscope (Zeiss) with a 100×/1.3 or 60×/1.3 Plan-Neofluar oil objective or a Nikon Ti2-eclipse fluorescence microscope (Nikon) with a 60× Plan Apo 1.4 NA oil objective. Images were analyzed using Fiji software (Version 2.1).

### Antibodies

Anti-importin β (57), anti-CRM1 (58), and anti-importin 13 (18) antibodies were described previously. Anti-MBP (New England Biolabs, E8032S), anti-NOSIP (Sigma Aldrich, HPA062132), anti-importin 7 (Thermo Fisher Scientific, PA5-115423), anti-importin α (Merck Millipore, 05–1526), anti-TNPO1 (Sigma Aldrich, # T0825, clone D45), anti-HA (Sigma Aldrich, H6908), and anti-tubulin (ProteinTech, 11224-1-AP) are commercially available. Alexa Fluor secondary antibodies for immunofluorescence were obtained from Thermo Fisher Scientific. IRDye 800CW, IRDye 680CW, or IRDye 680LT (LI-COR) were used as secondary antibodies for Western blotting.

### Heterokaryon assay

To analyze shuttling of NOSIP, 50,000 HeLa cells were seeded on coverslips in 24-well plates and transfected with plasmids coding for NOSIP-HA or GFP-GST-NOSIP or control construct Rev(47–116)-GFP_2_-cNLS. Transfected HeLa cells were cocultured with NIH3T3 cells and treated with cycloheximide (100 μg/μl) to stop protein translation in the presence (+LMB) or absence (-LMB) of 10 nM LMB for 3 h at 37 °C and 5% CO_2_. Cells were fused using 50% PEG 2000 for 3 min at RT. After four extensive washing steps with PBS, fused cells were incubated in DMEM containing cycloheximide (Sigma Aldrich, C7698, 100 μg/μl) for 3 h in the presence or absence of LMB (10 nM), fixed with 3.7% formaldehyde in PBS and analyzed by indirect immunofluorescence. Cells were analyzed by confocal microscopy using an LSM 510-META confocal laser scanning microscope (Zeiss) with a 100× Plan-Neofluar 1.3 oil objective.

### Nuclear transport assay in living cells (glucocorticoid receptor assay)

HeLa cells were seeded in 24-well plates on coverslips and transfected with plasmids coding for GR_2_-GFP-NOSIP or Rev-GR-GFP. Import of GFP-reporter proteins was induced by adding 5 μM dexamethasone (Calbiochem) in DMEM containing 100 μg/μl cycloheximide for 1 h at 37 °C. Cells were washed three times with PBS and fixed in 3.7% formaldehyde for 10 min. For analysis of nuclear export, import was induced as described above. After three washing steps with PBS, cells were incubated with fresh DMEM containing cycloheximide (100 μg/μl) and lacking dexamethasone for 2 h at 37 °C, washed again 3 times with PBS, fixed in 3.7% formaldehyde in PBS for 10 min, and mounted in Mowiol containing DAPI. Cells were analyzed by fluorescence microscopy using a Nikon Ti-2 eclipse with a 60× Plan Apo 1.4 NA oil objective.

### Nuclear import assay *in vitro*

Nuclear import reactions in permeabilized cells were essentially performed as described (59). Briefly, HeLa cells grown on poly-L-lysine (Sigma Aldrich)-coated coverslips were permeabilized with 0.005% digitonin in TPB containing 1 μg/ml each of leupeptin, pepstatin, and aprotinin for 5 min on ice. Transport reactions contained an ATP-regenerating system (1 mM ATP, 5 mM creatine phosphate, 20 U/ml creatine phosphokinase), 2 mg/ml BSA, 500 nM His-NOSIP-MBP, 4 μM RanWT, and 1 μM purified NTR (His-Importin 13, His-TNPO1, His-Importin 7, His-Importin β/7, His-importin β, or His-Importin α/β) or 1.4 mg/ml cytosol in a total volume of 40 μl. Reactions were incubated for 30 min at 30 °C or 4 °C in a humidity chamber, followed by three washing steps for 3 min each with ice-cold TPB and fixation with 3.7% formaldehyde in TPB for 10 min at 4 °C. His-NOSIP-MBP was visualized by indirect immunofluorescence with an antibody against MBP. After immunostaining, cells were mounted in Mowiol containing DAPI and analyzed by confocal microscopy using a 100× Plan-Neofluar 1.3 NA oil objective. Images were processed using Fiji and cell profiler (60) (version 4.2.1.).

### Image analysis

Microscopy images were analyzed using CellProfiler software (61). Cell nuclei were identified by DAPI staining using Otsu thresholding. The whole cell region was defined by using the identified nuclei as a seed region and expanding it depending on the intensity using the minimum cross entropy method and a minimum intensity of 0.01. To obtain the cytoplasmic region, the nuclear region was subtracted from the cell region. Afterward, either cotransfected cells were filtered for signals in the 488 nm and 594 nm channels or single transfected cells were only filtered for the signal in the 488 nm channel, for a minimum intensity of 0.01. Nuclear and cytoplasmic fluorescence intensities were then measured in the 488-nm channel and the nuclear/cytoplasmic ratio (N/C) was calculated. For every condition, 500 to 1000 cells were analyzed and data were plotted using GraphPad Prism 9. For statistical analysis, one-way ANOVA was performed, followed by Bonferroni posttest, considering *p*-values <0.05 as significant (∗: ≤0.05, ∗∗: ≤0.01, ∗∗∗: ≤0.001).

For the analysis of *in vitro* transport assays, nuclei were identified using DAPI staining and shrunken by 10 pixels to measure fluorescence intensities only in the nucleus. Afterward, the cell regions were identified as described above and cells were filtered for a minimum fluorescence intensity of 0.01 in the 488 channel and the mean fluorescence intensity was measured. The mean values of a 4 °C control were subtracted as background from the mean values of the respective 37 °C samples. This corrected mean fluorescence intensity was normalized to a reaction containing cytosol, which was arbitrarily set to 1. Data were plotted in GraphPad Prism 9.

### Cross-linking and mass spectrometric analysis

His-NOSIP, 20 μM, and 20 μM His-TNPO1 were incubated for 2 h on ice in a total volume of 300 μl, and complexes were purified using a Superdex S200 analytical 10/300 GL column (Cytiva) equilibrated in cross-linking buffer (20 mM Na_2_HPO_4_/NaH_2_PO_4_ pH 8.0, 150 mM NaCl, 1 mM EDTA, and 5% glycerol). Complex-containing fractions were pooled and cross-linked using bis(sulfosuccinimidyl)suberate (BS3, Thermo Fisher, A39266, dissolved in dimethyl sulfoxide) in 300-fold molar excess for 30 min at RT. Reactions were stopped by adding Tris pH 8.0 to a final concentration of 50 mM. Cross-linked complexes were subjected to SDS-PAGE (NuPAGE gradient 4–12%), and shifted bands in the gel corresponding to TNPO1 cross-linked to NOSIP were cut out and analyzed as described (62). Briefly, proteins were digested with trypsin at a 1:20 (w/w) enzyme to substrate ratio at RT overnight. Samples were dried in a vacuum centrifuge and subsequently, dried peptides were reconstituted in 30% (v/v) acetonitrile, 0.1% (v/v) trifluoroacetic acid and fractionated by peptide size-exclusion chromatography as described elsewhere (63). Fractions containing cross-linked peptides were dried again in a vacuum centrifuge.

LC-MS analysis was performed on a Q Exactive HF or an Orbitrap Exploris 480 mass Spectrometer coupled to a Dionex UltiMate 3000 RSLCnano system (both Thermo Fisher Scientific). Dried peptides were resuspended in 5% (v/v) acetonitrile, 0.1% trifluoroacetic acid (v/v) and separated on a C18 PepMap100 μ-Precolumn (0.3 × 5 mm, 5 μm, Thermo Fisher Scientific) followed by an in-house packed main column (75 μm × 30 cm, Reprosil-Pur 120C18-AQ, 1.9 μm, Dr Maisch GmbH) at 300 nl/min flow rate. The gradient for in-gel digested samples was set to 37 min from 10% to 36% buffer B (80% (v/v) acetonitrile, 0.08% (v/v) formic acid) followed by an increase to 45% buffer B for 6 min with an overall method duration of 58 min.

MS1 scans were acquired with 120,000 resolution (full width at half maximum), 1 × 10^6^ automatic gain control target, and 25 ms maximum injection time from 350 to 1600 *m/z* scan range. The 25 most abundant precursor ions were selected individually with a 1.4 *m/z* isolation window and were fragmented with a normalized collision energy of 30. MS2 scans were acquired with 30,000 resolution full width at half maximum, 1 × 10^5^ target automatic gain control, 120 ms maximum injection time, and a fixed first mass of 110 *m/z*. Precursors with a charge smaller than 3 and larger than 8 were excluded from isolation and fragmentation, and a dynamic exclusion was set to 10 s.

### Database search

Acquisition files were subjected to database searching with pLink version 2.3.9 (64) against the protein sequences of TNPO1 and His-NOSIP and common contaminants. BS3 was set as a cross-linker, carbamidomethylation on cysteine was set as fixed, and oxidation on methionine was set as variable modification. Tryptic cleavage specificity with up to three missed cleavages or chymotryptic cleavage specificity with up to five missed cleavages was considered for peptides between 6 and 60 amino acids length and 600 to 6000 Da size. The peptide-, fragment-, and filter-tolerance were set to 20, 20, and 10 ppm, respectively. A 5% false discovery rate cutoff on spectral level was applied separately for inter- and intraprotein cross-links. Identifications were further filtered for at least four fragment ions for each of the peptides in a cross-link pair, a minimum score of 5, and more than 2 spectrum identifications.

## Data availability

The mass spectrometry proteomics data (datasets “Transportin_lower_bands [1–3] [a/b]” and the respective analysis files) have been deposited to the ProteomeXchange Consortium *via* the PRIDE (65) partner repository with the dataset identifier PXD033966 reviewer_pxd033966@ebi.ac.uk. All other data are contained within the article.

## Supporting information

This article contains supporting information (24).

## Conflict of interest

The authors declare that they have no conflicts of interest with the contents of this article.
