# Supplementary material for: Transportin 1 is a major nuclear import receptor of the nitric oxide synthase interacting protein
Source: J Biol Chem. 2023 Jan 20;299(3):102932. doi: 10.1016/j.jbc.2023.102932 (PMC9974451; doi:10.1016/j.jbc.2023.102932)
Supplement: Supplemental Table S1 [file mmc1.pdf]

Table S1: Identified inter-protein crosslinks of Transportin 1-NOSIP complexes crosslinked by BS3

Protein 1 (Name|UniprotID)	Protein 2 (Name|UniprotID)	residue 1	residue 2	Score	Spectral Count
NOSIP_human|Q9Y314	Transportin_human|Q92973	157	197	5,480	4
NOSIP_human|Q9Y314	Transportin_human|Q92973	178	81	6,123	10
NOSIP_human|Q9Y314	Transportin_human|Q92973	132	197	6,561	9
NOSIP_human|Q9Y314	Transportin_human|Q92973	117	197	7,103	26
NOSIP_human|Q9Y314	Transportin_human|Q92973	153	81	8,409	23
NOSIP_human|Q9Y314	Transportin_human|Q92973	100	81	8,629	157
NOSIP_human|Q9Y314	Transportin_human|Q92973	90	128	8,871	8
NOSIP_human|Q9Y314	Transportin_human|Q92973	178	502	8,915	9
NOSIP_human|Q9Y314	Transportin_human|Q92973	175	81	9,310	116
NOSIP_human|Q9Y314	Transportin_human|Q92973	132	81	9,331	33
NOSIP_human|Q9Y314	Transportin_human|Q92973	289	81	9,570	5
NOSIP_human|Q9Y314	Transportin_human|Q92973	155	81	9,775	24
NOSIP_human|Q9Y314	Transportin_human|Q92973	178	385	10,945	70
NOSIP_human|Q9Y314	Transportin_human|Q92973	21	889	10,968	8
NOSIP_human|Q9Y314	Transportin_human|Q92973	172	85	11,654	17
NOSIP_human|Q9Y314	Transportin_human|Q92973	117	66	11,755	20
NOSIP_human|Q9Y314	Transportin_human|Q92973	90	85	11,967	20
NOSIP_human|Q9Y314	Transportin_human|Q92973	132	66	12,813	6
NOSIP_human|Q9Y314	Transportin_human|Q92973	157	81	13,216	50
NOSIP_human|Q9Y314	Transportin_human|Q92973	172	81	15,279	28
NOSIP_human|Q9Y314	Transportin_human|Q92973	21	81	15,261	31
NOSIP_human|Q9Y314	Transportin_human|Q92973	175	85	16,425	42
NOSIP_human|Q9Y314	Transportin_human|Q92973	117	81	20,111	67
NOSIP_human|Q9Y314	Transportin_human|Q92973	175	128	13,916	12

Raw data (datasets “Transportin_lower_bands [1-3] [a/b]” (a and b correspond to the technical replicates) and the respective analysis files) have been deposited to the ProteomeXchange Consortium via the PRIDE partner repository with the dataset identifier PXD033966. Interprotein crosslinks were filtered for minimum score of 5 and >2 spectra identifications. For details see *Experimental Procedures.*
