# Supplementary material for: Transportin 1 is a major nuclear import receptor of the nitric oxide synthase interacting protein
Source: J Biol Chem. 2023 Jan 20;299(3):102932. doi: 10.1016/j.jbc.2023.102932 (PMC9974451; doi:10.1016/j.jbc.2023.102932)
Supplement: Supplemental Figure S1 [file mmc2.pdf]

Figure S1

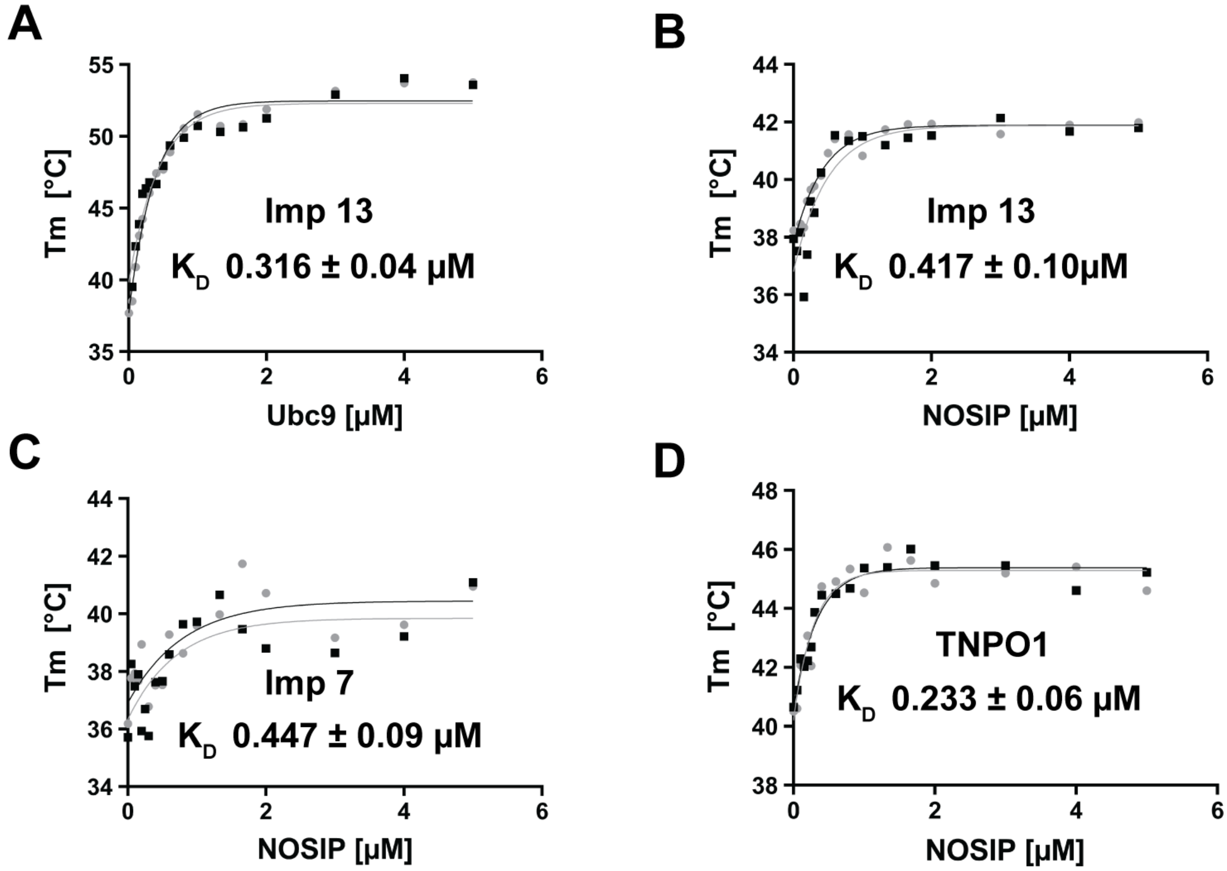

Fig. S1. Transportin has a high affinity for NOSIP.
K_D-values were determined by differential scanning fluorimetry (DSF). Increasing amounts of NOSIP (or Ubc9 in A, as indicated) were added to NTR (Imp 13 (A, B), Imp 7 (C) or TNPO1 (D)) and melting curves were measured. The melting temperature (Tm) of each step was calculated by fitting the Boltzmann equation to each individual melting curve. The calculated melting temperatures were then plotted against the concentration of NOSIP (or Ubc9). The resulting curve was fitted with an exponential equation to determine dissociation constants. For each NTR, 3-4 independent experiments were performed and data for two characteristic experiments are depicted. The measured K_D of Ubc9 and importin 13 was very similar to that determined previously (Grünwald et al., 2013, reference 24) using the same method. Note that no meaningful curves were obtained when NTR were omitted from the reaction or replaced by BSA, an inert control protein (data not shown).
