# Supplementary material for: Transportin 1 is a major nuclear import receptor of the nitric oxide synthase interacting protein
Source: J Biol Chem. 2023 Jan 20;299(3):102932. doi: 10.1016/j.jbc.2023.102932 (PMC9974451; doi:10.1016/j.jbc.2023.102932)
Supplement: Supplemental Figure S2 [file mmc3.pdf]

# Figure S2

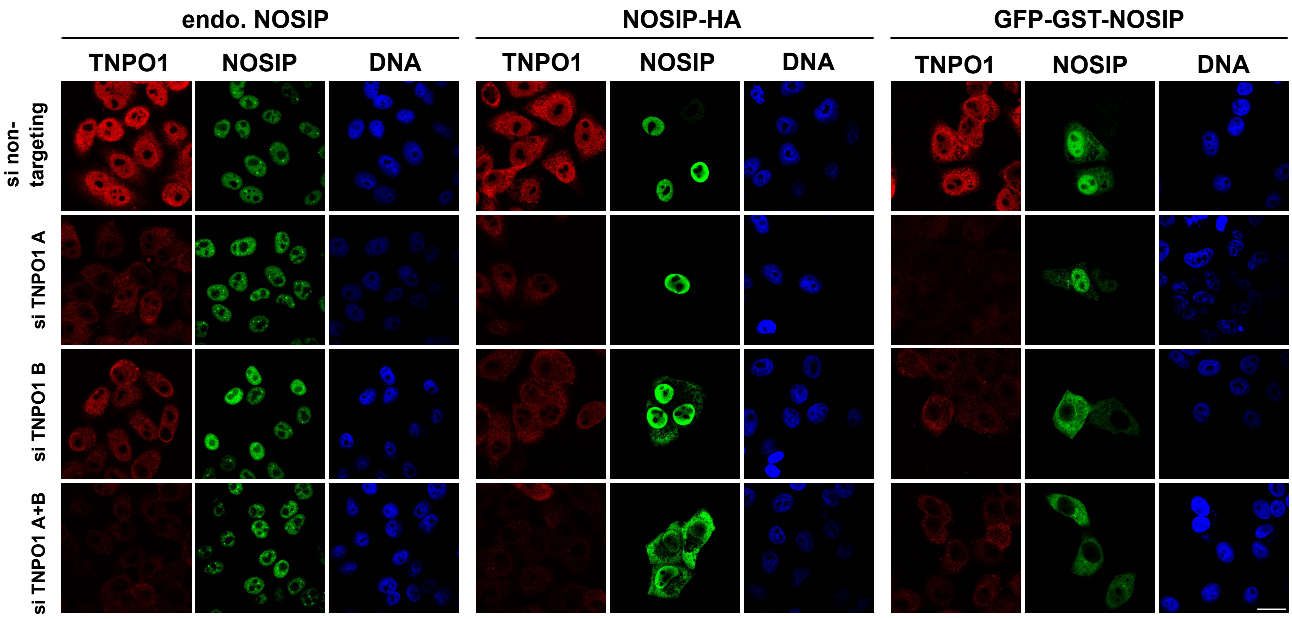


**Fig. S2.** Transportin is a major import receptor for NOSIP.
Cells were cotransfected with plasmids coding for HA-or GFP-GST-tagged NOSIP and with control siRNAs or siRNAS against TNPO1 (si RNAs A and B, as indicated. Endogenous (endog.) or overexpressed NOSIP (green) was visualized by indirect immunofluorescence with anti-HA or anti-NOSIP antibodies or directly via the GFP-tag. Nuclei were stained with DAPI (blue) and cells were analyzed by confocal microscopy. Scale bar, 20 µm. Compare Fig. 6D.
